# Synthesis and Biological Evaluation of 4-Aroyl-6,7,8-Trimethoxyquinolines as a Novel Class of Anticancer Agents 

**DOI:** 10.3390/molecules16032274

**Published:** 2011-03-07

**Authors:** Cheng-Chih Hsieh, Hsueh-Yun Lee, Chih-Ying Nien, Ching-Chuan Kuo, Chi-Yen Chang, Jang-Yang Chang, Jing-Ping Liou

**Affiliations:** 1 Graduate Institute of Medical Sciences, National Defense Medical Center, No.161, Sec.6. Min-Chuan E. Road, Taipei 114, Taiwan; 2 Department of Pharmacy Practice, Tri-Service General Hospital, No.325, Sec.2. Cheng-Kung Road, Taipei 114, Taiwan; 3 School of Pharmacy, College of Pharmacy, Taipei Medical University, No. 250 Wuxing Street, Taipei 11031, Taiwan; 4 National Institute of Cancer Research, National Health Research Institutes. 2 F, No.367, Sheng Li Road, Tainan 704, Taiwan; E-Mails: cckuo@nhri.org.tw (C.-C.K.); chiyen1215@nhri.org.tw (C.-Y.C.)

**Keywords:** combretastatin analogs, multidrug-resistant, trimethoxyquinolines, antiproliferative activity

## Abstract

A series of 2-aroyl and 2-aryl-5,6,7-trimethoxyquinoline and 4-aroyl-6,7,8-trimethoxyquinoline combretastatin analogs were synthesized and evaluated for their potential anticancer activity. The 4-aroylquinoline **11** inhibited the growth of the human cancer cells lines KB, HT-29, and MKN45, as well as the three human resistant cancer cell lines KB-vin10, KB-S15, and KB-7D, with IC_50_ values of 217, 327, 239, 246, 213, and 252 nM, respectively.

## 1. Introduction

**Figure 1:**
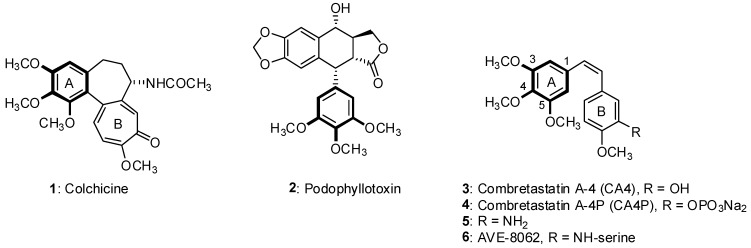
Natural tubulin polymerization inhibitors

From past to present natural products have directly or indirectly contributed to the discovery of approximately 50% of modern drugs [1]. Due to natural products’ structural diversity and potent biological activity, their novel scaffolds provide opportunities for scientists in the development of numerous biologically active products as well [2]. The mitotic process of the division step of the cell cycle involves the assembly of microtubules. Since the dynamic structures of microtubules play a crucial role in cellular division, they are recognized as an important target for anticancer therapy [3]. Several natural products have been identified as potent antiproliferative agents, for instance, colchicine (**1**), podophyllotoxin (**2**) [4] and combretastatin A-4 (CA4, **3**) [5] (Figure 1). The main mechanism of the above products was shown to involve interaction with microtubules at the colchicine binding site leading to an M-phase arrest. Among them, CA4 has attracted lots of effort in the development of antimitotic agents owing to its distinct structural characteristics. For instance, the phosphate prodrug CA4P (**4**; Figure 1) with improved solubility is in Phase II/III clinical trials [6]. Additionally, compound **5**, where the 3’-OH is replaced with a 3’-NH_2_ has also demonstrated potent antitubulin activity [7] and its serine prodrug (AVE-8062, **6**, Figure 1) is undergoing phase III clinical trials as well. 

**Figure 2:**
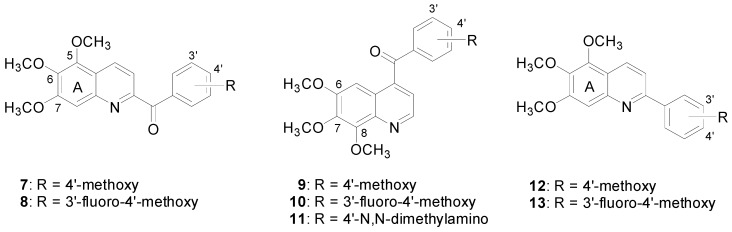
Synthetic 4-aroyl-6,7,8-trimethoxyquinolines,2-aroyl- and 2-aryl-5,6,7-trimethoxyquinolines.

When these three natural products are compared, the trimethoxybenzene moiety is seen to play a crucial role in their potent biological activity. In a previous study we synthesized a series of 2-amino-benzophenones containing 3,4,5-trimethoxybenzene moieties, and those synthesized products revealed potent antiproliferative activity, comparable to that of CA4 [8,9]. In the structural analysis of colchicine, the [6,7,7]-tricyclic colchicine unit possessing a trimethoxyphenyl motif attracted our interest to investigate the effect of trimethoxy-substituted heterocycles on the antiproliferative activity. On the basis of this motivation, in this study and based on our prior experience [10] we planned to select the quinoline moiety as the core structure. As a result, a series of 6,7,8-trimethoxyquinolines and 5,6,7-trimethoxyquinolines were synthesized as potential antiproliferative agents (Figure 2).

## 2. Results and Discussion

**Scheme 1:**
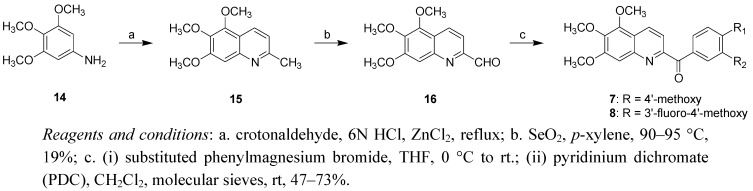
Synthetic route to 2-aroyl-5,6,7-trimethoxyquinolines **7** and **8**.

First, we introduced the aroyl substituents on the C-4 of 6,7,8-trimethoxyquinoline and C-2 of 5,6,7-trimethoxyquinoline, respectively. The synthesis of the 2-aroyl-5,6,7-trimethoxyquinolines is illustrated in Scheme 1. The commercially **14** underwent a Doebner-von Miller reaction with crotonaldehyde to afford 2-methyl-5,6,7-trimethoxyquinoline (**15**). Subsequently, benzylic oxidization was effected by the treatment with selenium dioxide to afford the aldehyde **16**. Compound **16** was reacted with appropriate substituted phenylmagnesium bromides followed by oxidation with pyridinium dichromate to yield the series of 2-aroyl-5,6,7-trimethylquinolines **7** and **8**. 

**Scheme 2:**
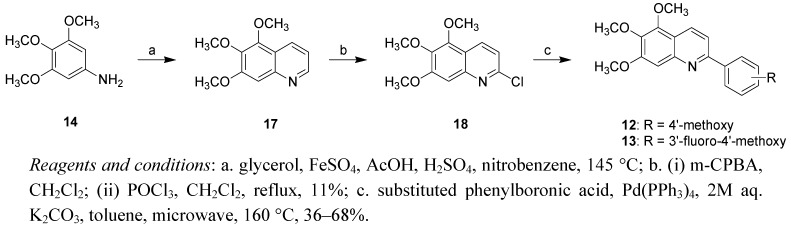
Synthetic route to 2-aryl-5,6,7-trimethoxyquinolines **12** and **13**.

In addition, the 2-phenyl-5,6,7-trimethoxyquinoloines were synthesized as well (Scheme 2) in order to investigate the impact of a carbonyl linkage between the quinoline and phenyl moieties. The Skraup quinoline synthesis was performed by the reaction between compound **14** and glycerol to provide the 5,6,7-trimethoxyquinoline (**17**). Compound **17** underwent chlorination by reacting with m-CPBA and POCl_3_ to afford compound **18**, followed by reaction of the latter with 4-methoxy-phenylboronic acid or 3-fluoro-4-methoxyphenylboronic acid to give compounds **12** and **13**, respectively.

**Scheme 3:**
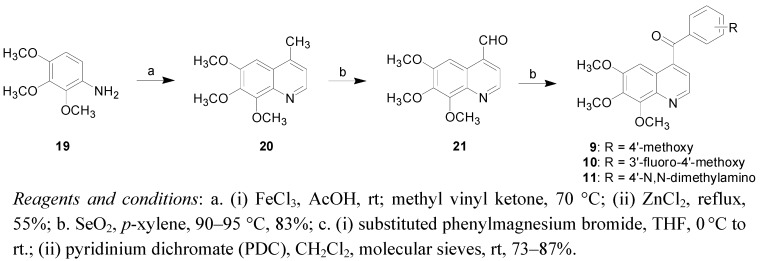
Synthetic route to 4-aroyl-6,7,8-trimethoxyquinolines **9**–**11**.

Scheme 3 depicts the synthesis of the 4-aroyl-6,7,8-trimethoxyquinolines. 4-Methyl-6,7,8-tri-methoxyquinoline (**20**) was obtained through the reaction between **19** and methyl vinyl ketone in the presence of ferric chloride and acetic acid. The subsequent benzylic oxidation was carried out with selenium dioxide to afford the corresponding aldehyde **21**. Compound **21** was then reacted with substituted phenylmagnesium bromides followed by oxidation with pyridinium dichromate to yield the 4-aroyl-6,7,8-trimethoxyquinolines **9**–**11**.

To evaluate the effect of the various trimethoxy-substituted heterocycles on the cancer cell inhibitory ability, the synthesized 2-aroyl-5,6,7-trimethoxyquinolines **7**, **8**, 4-aroyl-6,7,8-trimethoxy-quinolines **9**–**11**, 2-aryl-5,6,7-trimethoxyquinolines **12**, **13**, and reference compound colchicine were evaluated for their antiproliferative activities against three human cancer cell lines, cervical carcinoma KB cells, colorectal carcinoma HT29 cells, and stomach carcinoma MKN45 cells (Table 1). Our previous study on the aminobenzophenone analogues demonstrated that the utilization of various aroyl substituents contributed to the improvement of antiproliferative activity [8,11]. Herein some of those aroyl functionalities, 4’-methoxybenzoyl, 3’-fluoro-4’-methoxybenzoyl and *N,N*-dimethylamin-obenzoyl, were applied and linked with 5,6,7-trimethoxyquinolines and 6,7,8-trimethoxyquinolines, respectively. The biological examination of 5,6,7-trimethoxyquinolines did not show antiproliferative activity, no matter whether they bore 2-aroyl or 2-aryl groups. This suggested that the 5,6,7-trimethoxyquinoline scaffold was unfavourable. In the 6,7,8-trimethoxyquinoline series, all compounds revealed better antiproliferative ability as compared with the 5,6,7-trimethoxyquinoline derivatives. Compound **11** showed the most potent activity. The addition of fluorine at the C-3’ position contributed a slight increase of activity, evident when **10 **is compared with **9**. The potency was improved about 6-fold after the 4’-methoxy was replaced with a 4’-*N,N*-dimethylamino functionality in compound **11**. Compound **11** inhibited the growth of KB, HT29 and MKN45 cancer cell lines with IC_50_ values of 217, 327 and 239 nM, respectively.

**Table 1:** IC_50_ Values (nM ± SD^a ^) of Compounds **7**–**13**.

	Cell Type ( IC_50 _± SD^a^, nM)								
Compd	KB			HT29			MKN45		
**7**	> 5,000			> 5,000			> 5,000		
**8**	> 5,000			> 5,000			> 5,000		
**9**	1,297	±	371	2,092	±	281	1,436	±	272
**10**	1,597	±	94	1,725	±	530	1,047	±	87
**11**	217	±	39	327	±	93	239	±	47
**12**	> 5,000			> 5,000 > 5,000			> 5,000		
	> 5,000						**14**		
	> 5,000						> 5,000		
**13**	> 5,000			> 5,000 > 5,000			> 5,000		
	> 5,000						**15**		
	> 5,000						> 5,000		
**colchicine**	10.3	±	0.9	16.2	±	2.9	19.2	±	1.0

^a^SD: standard deviation, all experiments were independently performed at least three times.

In an effort to further understand the efficacy of 4-aroyl-6,7,8-trimethoxyquinoline against drug-resistant cell lines, compound **11** was evaluated for antiproliferative activity against various resistant lines, as shown in the Table 2. Despite the high level of expression drug-resistant efflux protein (MDR/P-gp or MRP) in KB-Vin10, KB-S15, and KB-7D cells, compound **11** showed similar cytotoxic efficacy towards these resistant lines and their parental cells.

**Table 2:** Growth inhibition of compound **11** and reference compoundsagainst drug-resistant cell lines.

Cell lines	Resistant type	IC_50 _± SD*^a^*			
		Vincristine, nM*^b^*	Paclitaxel, nM*^b^*	VP-16, µM*^b^*	11, nM
KB	Parental	0.4 ± 0.1	3.3 ± 1.2	1.1 ± 0.5	217.0 ± 39.0
KB-VIN10	MDR ↑	90.1 ± 7.4	16500 ± 707	23 ± 3	245.6 ± 8.5
KB-S15	MDR ↑	17.6 ± 2.2	273 ± 15	3.5 ± 0.3	213.3 ± 96.1
KB-7D	MRP ↑	1.2 ± 0.4	7.9 ± 0.5	54 ± 3.5	252.3 ± 15.0

*^a^*SD: standard deviation, all experiments were independently performed at least three times; *^b^*Data is from ref. [12].

As mentioned above, the main mechanism of action of the trimethoxyphenyl-containing natural products shown in Figure 1 was through to involve interaction with microtubules at the colchicine binding site leading to M-phase arrest. To investigate whether the activities of 4-aroyl-6,7,8-trimethoxyquinoline derivatives were related to interactions with the microtubule system, compounds **11** and the reference compound colchicine were evaluated for inhibition of tubulin polymerization activity and colchicine binding activity. Table 3 shows that compound **11** was only slightly bound to the colchicine binding site, however, it was less potent in tubulin polymerization. This result suggested that compound **11** may inhibit cancer cell growth through an alternative mechanism and this needs further research. 

**Table 3:** Inhibition of tubulin polymerization and colchicine binding by compound **11** and colchicine.

Compd.	Tubulin*^a^*IC_50_ ± SD (µM)	Colchicine binding*^b^* (%)	
		1 µM	5 µM
**11**	>10	37.6	45.6
**c** **olchicine**	7.3	58.7	80.8

*^a^* Inhibition of tubulin polymerization [13]; *^b^* Inhibition of [^3^H] colchicine binding. Tubulin was at 1 µM; both [^3^H]colchicine was at 5 µM.

## 3. Experimental

### 3.1. General

Nuclear magnetic resonance (^1^H-NMR) spectra were obtained on a Bruker DRX-500 spectrometer (operating at 500 MHz). Chemical shifts are reported in parts per million (ppm, *δ*) downfield from TMS used as an internal standard. High-resolution mass spectra (HRMS) were measured with a JEOL (JMS-700) electron impact (EI) mass spectrometer. Flash column chromatography was done using silica gel (Merck Kieselgel 60, No. 9385, 230-400 mesh ASTM). All reactions were carried out under an atmosphere of dry nitrogen.

### 3.2. Chemistry

*5,6,7-Trimethoxyquinoline-2-carboxaldehyde* (**16**). To a warm solution of 3,4,5-trimethoxyaniline (5.0 g, 27.3 mmol) in 6 N HCl (35 mL) was added crotonaldehyde (2.0 g, 28.6 mmol) dropwise, and the solution was refluxed for 1 hour. After cooling to room temperature, ZnCl_2_ (3.72 g, 27.3 mmol) was added and the mixture was refluxed for 4 hours, then ice was added. The dark viscous oil was extracted with NaHCO_3(aq)_ and CH_2_Cl_2_. The combined organic layer was dried over anhydrous MgSO_4 _and concentrated under reduced pressure to give a residue which was further treated with *p*-xylene (89 mL) and selenium dioxide (6.1 g, 21.4 mmol) at 90–95 °C overnight. The reaction mixture was filtered through a pad of Celite. The filtrate was evaporated to give a residue that was purified by silica gel flash column chromatography (*n*-hexane/ethyl acetate = 5:1) to afford **16**, yield 19%. ^1^H-NMR (CDCl_3_): *δ* 4.03 (s, 3H), 4.05 (s, 3H), 4.07 (s, 3H), 7.37 (s, 1H), 7.90 (d, *J* = 8.4 Hz, 1H), 8.50 (d, *J* = 8.4 Hz, 1H), 10.17 (s, 1H).

*6,7,8-Trimethoxy-4-methylquinoline* (**20**). To a stirred solution of 2,3,4-trimethoxyaniline (1.0 g, 5.46 mmol) in acetic acid (6.8 mL), ferric chloride (0.89 g, 5.46 mmol) was added. The reaction mixture was stirred for 5 minutes and then methyl vinyl ketone (0.52 mL, 6.0 mmol) was added slowly over a period of 15 minutes. The reaction mixture was heated to 70 °C for one hour. Anhydrous zinc chloride (0.74 g. 5.46 mmol) was added and the reaction was further refluxed for 16 hours. The reaction mixture was cooled, filtered, made basic with 10% NaOH solution, extracted with ethyl acetate (20 mL × 3), dried over Na_2_SO_4_ and evaporated to give the product **20**, yield 55%. ^1^H-NMR (CDCl_3_): *δ* 2.64 (s, 3H), 3.89 (s, 3H), 3.97 (s, 3H), 4.18 (s, 3H), 6.97 (s, 1H), 7.18 (d, *J* = 4.3 Hz, 1H), 8.68 (d, *J* = 4.4 Hz, 1H).

*6,7,8-Trimethoxyquinoline-4-carbaldehyde* (**21**). A mixture of **20** (0.5 g, 2.14 mmol), *p*-xylene (20 mL) and selenium dioxide (0.25 g, 2.23 mmol) was heated at 90–95 °C overnight. The reaction mixture was filtered through a pad of Celite. The filtrate was evaporated to give a residue that was purified by silica gel flash column chromatography (*n*-hexane/ethyl acetate = 5:1) to give **21**, yield 83%. ^1^H NMR (500 MHz, CDCl_3_): *δ* 4.05 (s, 3H), 4.06 (s, 3H), 4.16 (s, 3H), 7.70 (d, *J* = 4.3 Hz, 1H), 8.31 (s, 1H), 9.07 (d, *J* = 4.3 Hz, 1H), 10.37 (s, 1H).

#### 3.2.1. General procedure for synthesis of compounds **7-11**

A solution of phenylmagnesium bromide (5.4 mL, 1.0 M in THF, prepared in advance) was added slowly to the corresponding quinolinecarboxaldehyde (0.96 g, 3.6 mmol) in tetrahydrofuran (5.4 mL) at 0 °C. The reaction mixture was warmed to room temperature, and stirring was continued for another 48 hours. A saturated NH_4_Cl solution was slowly added to hydrolyze the adduct at 0 °C, and extracted with EtOAc (15 mL × 2) and CH_2_Cl_2_ (15 mL × 2). The combined organic extracts was dried over MgSO_4_ and evaporated to give a crude residue, which was dissolved in CH_2_Cl_2_ (50 mL). Molecular sieves (4 Å, 2.7 g) and pyridinium dichromate (2.7 g, 7.2 mmol) were added to the reaction mixture with stirring at room temperature for 16 hours. The reaction mixture was filtered through a pad of Celite. The filtrate was evaporated to give a residue that was purified by silica gel flash column chromatography (*n*-hexane/ethyl acetate) to afford the desired compound.

*2-(4’-Methoxybenzoyl)-5,6,7-trimethoxyquinoline* (**7**). The title compound was obtained in 52% overall yield from 4-methoxyphenylmagnesium bromide and 5,6,7-trimethoxyquinoline-2-carboxaldehyde. m.p. 103–105 °C. ^1^H-NMR (CDCl_3_): *δ* 3.90 (s, 3H), 4.01 (s, 3H), 4.02 (s, 3H), 4.09 (s, 3H), 6.99 (d, *J* = 8.8 Hz, 2H), 7.32 (s, 1H),7.90 (d, *J* = 8.6 Hz, 1H), 8.22 (d, *J* = 8.8 Hz, 2H), 8.51 (d, *J* = 8.5 Hz, 1H). MS (EI) *m/z*: 353 (M^+^, 56%), 135 (100%). HRMS (EI) for C_20_H_19_NO_5_ (M^+^): calcd, 353.1263; found, 353.1266.

*2-(3’-Fluoro-4’-methoxybenzoyl)-5,6,7-trimethoxyquinoline* (**8**). The title compound was obtained in 47% overall yield from 3-fluoro-4-methoxyphenylmagnesium bromide and 5,6,7-trimethoxyquinoline-2-carboxaldehyde. m.p. 137–139 °C. ^1^H-NMR (CDCl_3_): *δ* 3.98 (s, 3H), 4.02 (s, 6H), 4.09 (s, 3H), 7.05 (t, *J* = 8.2 Hz, 1H), 7.32 (s, 1H), 7.93 (d, *J* = 8.6 Hz, 1H), 8.06–8.09 (m, 2H), 8.52 (d, *J* = 8.6 Hz, 1H). MS (EI) *m/z*: 371 (100%). HRMS (EI) for C_20_H_18_FNO_5_ (M^+^): calcd, 371.1169; found, 371.1170.

*4-(4’-Methoxybenzoyl)-6,7,8-trimethoxyquinoline* (**9**). The title compound was obtained in 78% overall yield from 4-methoxyphenylmagnesium bromide and 6,7,8-trimethoxyquinoline-4-carboxaldehyde. ^1^H-NMR (CDCl_3_): *δ* 3.83 (s, 3H), 3.88 (s, 3H), 4.04 (s, 3H), 4.18 (s, 3H), 6.94–6.96 (m, 3H), 7.30 (d, *J* = 4.4 Hz, 1H), 7.83 (d, *J* = 8.9 Hz, 2H), 8.88 (d, *J* = 4.4 Hz, 1H). MS (EI) *m/z*: 353 (100%). HRMS (EI) for C_20_H_19_NO_5_ (M^+^): calcd, 353.1263; found, 353.1263.

*4-(3’-Fluoro-4’-methoxybenzoyl)-6,7,8-trimethoxyquinoline* (**10**). The title compound was obtained in 73% overall yield from 3-fluoro-4-methoxyphenylmagnesium bromide and 6,7,8-trimethoxyquinoline-4-carboxaldehyde. m.p. 117–118 °C. ^1^H-NMR (CDCl_3_): *δ* 3.73 (s, 3H), 3.90 (s, 3H), 3.92 (s, 3H), 4.05 (s, 3H), 6.86 (s, 1H), 7.28 (s, 1H), 7.45 (s, 1H), 7.52 (s, 1H), 7.70 (s, 1H), 8.85 (s, 1H). MS (EI) *m/z*: 353 (100%). HRMS (EI) for C_20_H_19_NO_5_ (M^+^): calcd, 353.1263; found, 353.1263.

*4-[4’-(N,N-Dimethylamino)benzoyl]-6,7,8-trimethoxyquinoline* (**11**). The title compound was obtained in 87% overall yield from 4-(*N,N*-dimethyl)anilinomagnesium bromide and 6,7,8-trimethoxy-quinoline-4-carboxaldehyde. ^1^H-NMR (CDCl_3_): *δ* 3.09 (s, 6H), 3.83 (s, 3H), 4.04 (s, 3H), 4.18 (s, 3H), 6.65 (d, *J* = 9.0 Hz, 2H), 6.96 (s, 1H), 7.29 (d, *J* = 4.0 Hz, 1H), 7.75 (d, *J* = 9.0 Hz, 2H), 8.87 (d, *J* = 4.5 Hz, 1H). MS (EI) *m/z*: 338 (100%). HRMS (EI) for C_20_H_22_N_2_O_3_ (M^+^): calcd, 338.1630; found, 338.1629.

*2-Chloro-5,6,7-trimethoxyquinoline* (**18**). A round bottle was charged FeSO_4_ (4.60 g, 16.37 mmol), 3,4,5-trimethoxyaniline (1.0 g, 5.46 mmol), glycerol (6.5 mL, 88.42 mmol), conc. H_2_SO_4_ (4.4 mL) and nitrobenzene (4.1 mL), then glacial acetic acid (4.9 mL) was added and the mixture was heated to 145 °C for 6 hours, then ice was added. After steam distillation, the dark viscous oil was extracted with saturated NaHCO_3_ and CH_2_Cl_2_. The combined organic layer was dried over anhydrous MgSO_4 _and concentrated under reduced pressure to give a residue, which was further treated with dichloromethane (9 mL), *m*-chloroperbenzoic acid (0.99 g, 5.75 mmol) at room temperature overnight, washed with 10% sodium sulfite, saturated NaHCO_3_ and saturated NaCl, and worked up. To the residue, dichloromethane (13.8 mL) and phosphoryl chloride (2.6 mL) were added and the mixture was stirred to reflux overnight. The reaction mixture was concentrated *in vacuo*, and the residue was extracted with dichloromethane. The organic layers were combined and evaporated to give a residue, which was purified by flash chromatography (*n*-hexane/ethyl acetate = 7:1) to give **18**, yield 11%. ^1^H-NMR (CDCl_3_)*: δ* 3.96 (s, 3H), 3.98 (s, 3H), 4.05 (s, 3H), 7.16 (s, 1H), 7.23 (d, *J* = 8.6 Hz, 1H), 8.28 (d, *J* = 8.6 Hz, 1H).

*2-(4’-Methoxyphenyl)-5,6,7-trimethoxyquinoline* (**12**). In a 10 mL glass tube were placed a magnetic stir bar, 2-chloro-5,6,7-trimethoxyquinoline (0.10 g, 0.39 mmol), 4-methoxyphenylboronic acid (0.19 g, 1.18 mmol), tetrakis(triphenylphosphine)palladium (0.04 g, 0.04 mmol) and 2 M potassium carbonate (1.1 mL) and toluene (3 mL). The vessel was sealed and placed into the microwave cavity. The reaction mixture was held at 160 °C for 10 min. After it was cooled to room temperature, the mixture was poured into water and then extracted with EtOAc and NaHCO_3(aq)_. The organic layers were combined and evaporated to give a residue, which was purified by flash chromatography (*n*-hexane/ethyl acetate = 4:1) to give **12**, yield 65%. m.p. 136–137 °C. ^1^H-NMR (CDCl_3_): *δ* 3.88 (s, 3H), 3.99 (s, 3H), 4.02 (s, 3H), 4.07 (s, 3H), 7.03 (d, *J* = 8.7 Hz, 2H), 7.31 (s, 1H), 7.68 (d, *J* = 8.7 Hz, 1H), 8.08 (d, *J* = 8.7 Hz, 2H), 8.37 (d, *J* = 8.7 Hz, 1H). MS (EI) *m/z*: 325 (100%). HRMS (EI) for C_19_H_19_NO_4_ (M^+^): calcd, 325.1314; found, 325.1317.

*2-(3’-Fluoro-4’-methoxyphenyl)-5,6,7-trimethoxyquinoline* (**13**). The title compound was obtained in 36% overall yield from 2-chloro-5,6,7-trimethoxyquinoline and 3-fluoro-4-methoxyphenylboronic acid in a similar manner as described for the preparation of **12**. m.p. 129–130 °C. ^1^H-NMR (CDCl_3_):*δ* 3.96 (s, 3H), 3.99 (s, 3H), 4.03 (s, 3H), 4.07 (s, 3H), 7.07 (t, *J* = 8.5 Hz, 1H), 7.29 (s, 1H), 7.66 (d, *J* = 8.7 Hz, 1H), 7.85 (d, *J* = 8.4 Hz, 1H), 7.94 (dd, *J* = 1.9, 12.6 Hz, 1H), 8.38 (d, *J* = 8.7 Hz, 1H). MS (EI) *m/z*: 343 (100%). HRMS (EI) for C_19_H_18_FNO_4_ (M^+^): calcd, 343.1220; found, 343.1223.

### 3.3. Cell Growth Inhibitory Assay

Human oral epidermoid carcinoma KB cells, colorectal carcinoma HT29 cells, and stomach carcinoma MKN45 cells were maintained in RPMI-1640 medium supplied with 5% fetal bovine serum. KB-VIN10 cells were maintained in growth medium supplemented with 10 nM vincristine, generated from vincristine-driven selection, and displayed overexpression of P-gp170/MDR. Cell in logarithmic phase were cultured at a density of 5,000 cells/mL/well in a 24-well plate. KB-VIN10 cells were cultured in drug-free medium for 3 days prior to use. The cells were exposed to various concentrations of the test drugs for 72 h. The methylene blue dye assay was used to evaluate the effect of the test compounds on cell growth as described previously [14]. The IC_50_ value resulting from 50% inhibition of cell growth was calculated graphically as a comparison with the control. Compounds were examined in at least three independent experiments, and the values shown for these compounds are the mean and standard deviation of these data.

### 3.4. Tubulin Polymerization in Vitro Assay [13,15]

Turbidimetric assays of microtubules were performed as described by Bollag *et al*. [16] In brief, microtubule-associated protein (MAP)-rich tubulin (from bovine brain, Cytoskeleton, Denver, CO, USA) was dissolved in reaction buffer (100 mM PIPES (pH 6.9), 2 mM MgCl_2_, 1 mM GTP) to preparef 4 mg/mL tubulin solution. Tubulin solution (240 μg MAP-rich tubulin per well) was placed in 96-well microtiter plate in the presence of test compounds or 2% (v/v) DMSO as vehicle control. The increase in absorbance was measured at 350 nm in a PowerWave X Microplate Reader (BIO-TEK Instruments, Winooski, VT, USA) at 37 °C and recorded every 30 s for 30 min. The area under the curve (AUC) used to determine the concentration that inhibited tubulin polymerization to 50% (IC_50_). The AUC of the untreated control and 10 μM of colchicine was set to 100% and 0% polymerization, respectively, and the IC_50_ was calculated by nonlinear regression in at least three experiments.

### 3.5. Tubulin Competition-Binding Scintillation Proximity Assay [17,18,19]

This assay was performed in a 96-well plate. In brief, 0.08 (micro)M of [^3^H]colchicine was mixed with the test compound and 0.5 µg special long-chain biotin-labeled tubulin (0.5 µg) and then incubated in 100 µL of reaction buffer (80 mM PIPES, pH 6.8, 1 mM EGTA, 10% glycerol, 1 mM MgCl_2_, and 1 mM GTP) for 2h at 37 °C. Then 80 µg of streptavidin-labeled SPA beads were added to each reaction mixture. Then the radioactive counts were directly measured by a scintillation counter.

## 4. Conclusions

Based on the observation of several natural antimitotic products, the significant common trimethoxyphenyl moiety prompted us to design and synthesize a series of 5,6,7-trimethoxyquinolines and 6,7,8-trimethoxyquinolines. The biological results demonstrated that 4-aroyl-6,7,8-trimethoxyquinolines exhibited better antiproliferative activity. For example, compound **11** inhibited the human cancer cell growth of KB, HT-29, and MKN45, as well as the three human-resistant cancer cell lines KB-vin10, KB-S15, and KB-7D, with an IC_50_ of 217, 327, 239, 246, 213, and 252 nM, respectively. The lost of tubulin polymerization inhibitory activity and slight colchicines binding activity supposed that compound **11** may have an alternative or additional mechanism. In summary, the utilization of 6,7,8-trimethoxyquinoline as structural scaffold indeed could provide a direction toward further cancer cell growth inhibitors development.
